# Robotic-Assisted Radical Prostatectomy after the First Decade: Surgical Evolution or New Paradigm

**DOI:** 10.1155/2013/157379

**Published:** 2013-04-03

**Authors:** Douglas W. Skarecky

**Affiliations:** Department of Urology, University of California, Irvine, CA 92697, USA

## Abstract

Early studies indicate that robotic-assisted radical prostatectomy (RARP) has promising short-term outcomes; however, RARP is beyond its infancy, and the long-term report cards are now beginning. The important paradigm shift introduced by RARP is the *reevaluation of the entire open radical prostatectomy experience* in surgical technique by minimizing blood loss and complications, maximizing cancer free outcomes, and a renewed assault in preserving quality of life outcomes by many novel mechanisms. RARP provides a new technical “canvas” for surgical masters to create upon, and in ten years, has reinvigorated a 100-year-old “gold standard” surgery.

## 1. Introduction

The first successful report of a laparoscopic nephrectomy was in 1991 [1], and just one year later Schuessler et al. [2] reported the first laparoscopic radical prostatectomy (LRP). They later abandoned the surgery due to its difficulty and great length of time. The challenge of continued development of LRP moved across the Atlantic Ocean when in 1999 two groups in Paris reported fairly large promising series. In 1998, the group led by Dr. Guillonneau at the Institut Mutualiste Montsouris in Paris presented their initial experience with LRP [3] and led the way for other well-trained teams, to achieve comparable oncologic outcomes and perhaps improved functional outcomes, notably groups led by Dr. Jacob in France [4] and Dr. Rassweiler in Germany [5]. The technique was accepted in Europe but only embraced by a few centers in the United States.

 The laparoscopic experts in Europe were more readily able to overcome the learning curve of LRP and perform some of the more technically challenging aspects of LRP, such as the vesicourethral anastomosis, versus the experienced open but laparoscopically naïve surgeons in the United States. Thus, laparoscopy was quickly adopted and applied in Europe, but the technical and ergonomic challenges dampened the adoption of LRP over the vast open retropubic radical prostatectomy (RP) experience in North America. 

 To overcome these counterintuitive movements and limitations of vision of standard laparoscopy, newly developed robotic-assisted systems introduced magnified three-dimensional imaging, full-range motion surgical arms, articulating instruments with 7 degrees of freedom, and intuitive movements. Although originally designed for performing battlefield operations with the surgeon controlling the tele-manipulators in a console from a remote distance, robotic systems have been adapted in civilian hospitals and are effective in cardiac surgery and other medical procedures [6].

Beginning in 2000, several groups in Europe introduced urology to the da Vinci Surgical System (Intuitive Surgical, Sunnyvale, CA) with their initial reports of robotic-assisted laparoscopic radical prostatectomy (RARP) [7–9]. The first robot-assisted laparoscopic prostatectomy (RALP) was performed in 2000 [8], and now we have reached our first decade of experience. Robotic-assisted technology has generated much enthusiasm among urologists, particularly novice or nonlaparoscopically trained surgeons who can now transition to minimally invasive treatments. These groups had persevered and conquered the difficult learning curve of LRP paving the way for the rapid advancement of this technique. Interestingly, LRP remains more vigorous in Europe, with relatively small number of American centers tackling the problem. The group at Henry Ford Hospital sought to undertake LRP in the early 2000s, but their quest to establish a pure laparoscopic program stalled. Subsequently, they metamorphosed into the first large-scale robotic LRP program using the novel da Vinci robot [10]. Many approaches have propagated [11–17]. Although great number of RARP surgeons employ the transperitoneal approach, others have advocated for extraperitoneal, the anterior approach, or a posterior approach to the seminal vesicles (SVs). The distinction between an ascending, retrograde dissection and a descending, antegrade dissection is also debated [18].

Now, ten years beyond the first beginnings, laparoscopic robotic prostatectomy has experienced geometric growth in the United States. But robotic prostatectomy has brought new controversies in regard to costs, and whether the outcomes have improved to mitigate increased expenses. It is estimated that in 2013, over 80% of all radical prostatectomies performed in the United States will be done using the da Vinci robot; yet, accurate numbers have not been established. The 10-year experience is now being evaluated by consensus groups with regard to “Best Practices” and outcomes to fully appreciate the exploration and innovation of a new technique [21–24]. However, for RARP, many surgical and quality of life outcome topics still remain to be perfected to become a new surgical paradigm.

## 2. Overview of Operative Technical Steps

### 2.1. Indications/Contraindications

Standard indications and contraindications found in open and laparoscopic surgery persist for robot-assisted prostatectomy. Additionally, corticosteroid usage, small pubic spaces, and large body mass indices will provide increased challenges and specialized management. Tactile discrimination is diminished or absent but may be compensated by visualization by highly experienced RARP surgeons. 

### 2.2. Patient Selection

#### 2.2.1. Initial Learning Curve (1st 10–20 Cases)

 The “technical” learning curve is a series of benchmarks a surgeon must scale to completely master RARP [25, 26]. The initial yardstick was simply completing the surgery in a time competitive with open RP of 3-4 hours. Clearly, a long learning curve was associated with more complex oncologic outcomes of surgical margins, lymph node dissection, and QOL issues of continence and sexual function. As Herrel and Smith have reported, the learning curve to reduce positive surgical margins to a level comparable to open RP may take upwards of 150–250 cases [27]. One advantage of RARP is the extended use of case video recording and many worldwide conferences detailing these surgical advances, either by live cases or video lecture. Early pioneers of the technique have been willing to share their success to expand RARP and provided a wealth of training classes and information to allow second generation practitioners to “leapfrog” past early mistakes. In this sense, RARP rides the wave of a new paradigm for technical training and has been a key source in its worldwide acceptance.

Careful patient selection for the initial cases is strategic and instrumental to survive the learning curve of the first 10–20 cases. The early RARP cases should have no prior history of previous abdominal surgery (e.g., hernia repair) and avoid the challenges of large prostates and obese patients. Large prostates >60 cc's on ultrasound (U/S) are a challenge due to space limitations of the bony pelvis, and it is cognizant that transrectal U/S may underestimate prostate weight by 20% [28]. Likewise, an obese patient with a fatty bladder and/or rectosigmoid colon restricts the working space impairing vision and dissection. Avoid men with previous TURP, TUNA, and/or radiation therapy or men with previous hormone therapy. Large median lobes, chronic prostatitis, radiation therapy, or anticipated tissue scaring are also very troublesome for the inexperienced [21]. It is also generally recommended that normally potent men and lymph node dissections should be avoided in the first 10–20 cases.

#### 2.2.2. After Learning Curve

Although the learning curve for 4-hour surgical times may be accomplished in as few as 15 men, learning still persists (e.g., anastomosis, complications, and positive surgical margins) beyond these initial cases generally to 250 cases and incrementally beyond. Beyond the initial learning curve, the patient profile may be extended to obese patients, larger prostates, previous abdominal surgeries, RPLND, clinical T3 cancers, poor Gleason grades (8–10), or salvage prostatectomy [21]. With experience, it is easy to dissect men with prior mesh hernia repair, and the presence of an inguinal hernia is not a contraindication to robotic prostatectomy. Endoscopic repair of inguinal hernia is simple, and the insertion of mesh to cover the hernia takes less than 10 minutes [29]. It is not difficult to repair inguinal and umbilical hernias even in the learning curve.

### 2.3. Positioning and Preparation

**Figure 1:**
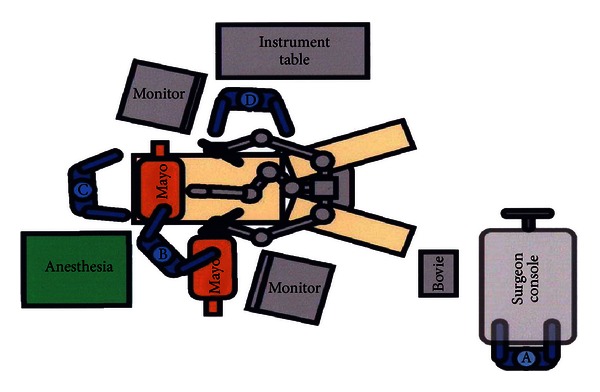
An example of a patient setup in an RARP operating theater [19].

It is important that all elements of the patient setup and the operating theater are consistent and reproducible, and an example is shown in Figure 1 [19]. The patient is placed supine on the operating room table that has been equipped with spreader bars. After general anesthesia is induced, the patient's arms are tucked at the side, and the legs are gently split and lowered to facilitate docking of the robot. It is imperative that before the patient is placed on the operating table, it is oriented correctly to allow maximum Trendelenburg position while keeping the head close to the floor. A Mayo stand is placed just over the patient's head and shoulders without interfering with the endotracheal tube. This Mayo is useful for instruments and protects the patient's head from the robotic camera and arms. An unusual problem is corneal abrasion brought on by the patient rubbing (and scratching) their eye due to positional edema. Our experience recommends that safety goggles should be placed over the patients eyes for about 90 minutes after the patient is extubated, and the goggles removed in recovery after the patient is fully awake.

### 2.4. Pneumoperitoneum and Trocar Placement

**Figure 2:**
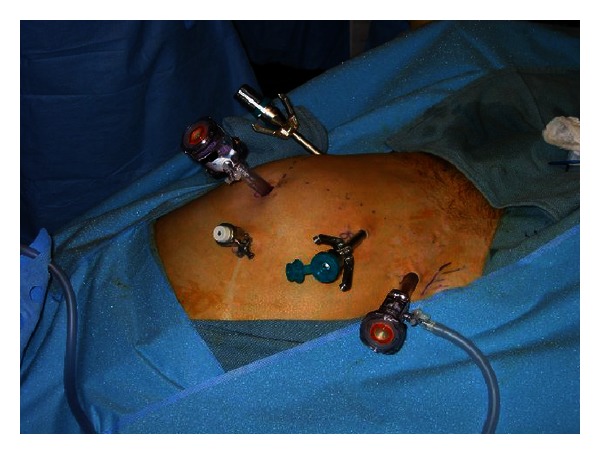
The camera port site is placed just above the navel, giving visual symmetry, easy removal of the prostate, and greater access, especially in older da Vinci models [20].

We previously highlighted the importance of the pubic symphysis and the navel in the location of port sites [20]. The camera port site is placed just above the navel, which allows for visual symmetry and easy removal of the prostate (Figure 2). Beck et al. recommends a transverse incision for the camera port rather than vertical in order to prevent postoperative incisional hernias [30]. In the older da Vinci system, the robotic arms have a maximum working length of 25 cms. The Pythagorean Theorem is used to calculate optimal port placement of the robotic arms (A); the arms should be within 18 cm of the pubic symphysis to reach the necessary depth of the working field. The robotic arms are introduced through 8 mm ports that are placed approximately one hand's breath (10 cm) from the camera port and no further than 18 cm from the symphysis pubis. The working distance is also reducible by 2-3 cm with pressure on the perineum, or the zero point of the da Vinci system can be adjusted by advancing the trocars 2-3 cm closer [20]. With the newer da Vinci S and Si robots, longer instrument lengths allow more flexibility in port placements, and the limitation on working length is no longer a significant issue. A transverse curvilinear incision at the perimeter of the umbilical crease is employed as this provides for better cosmesis at the skin level and a stronger fascial closure. An analysis of outcomes from our institution has demonstrated the benefits of the transverse incision beyond simple cosmesis to the significant reduction of primary hernias and reoperations to correct them, as opposed to the traditional vertical midline incision [30]. 

The other ports are placed, under direct vision. The assistant's dominant hand port (C) is a 5 mm port placed at least 6 cm lateral and cranial to the camera port. By necessity, left-handed assistants are on the patient's right side and the opposite is necessary for right-handed surgeons. This location allows the dominant hand of the assistant to be relatively free of the camera arm; however, because the port site is so cranial, it is necessary to use an extra long suction irrigation tip. Prostate sizes by transrectal ultrasound of <60–70 grams are recommended (Davol, Inc., Cranston, RI). The assistant's nondominant hand port (D) is a 12 mm port that is placed at least 6 cm lateral and inferior to the ipsilateral robotic arm. Through this port the assistant will use a locking grasper, a needle driver to pass and grasp suture, and an endoscopic vascular stapler for stapling the dorsal venous complex. With 4-arm systems, especially the S systems, 6 ports are used. The 6th port usually goes in a straight line on the opposite side of the 12 mm assistant port. 

### 2.5. The Operative Technical Steps (Box 1)

#### 2.5.1. Anterior Bladder Mobilization

(i)* Apical Dissection*

(a)* Early Technique. *Initially the dissection started with incision of the endopelvic fascia followed by dissection of the levator muscles freeing the prostatic apex. Fat overlying the puboprostatic ligaments was only partially dissected. Two figure of 8 sutures were placed, proximally and distally, to control the DVC. The prostate was completely freed following which the DVC and urethra were divided using electrocautery. This technique was problematic because a large bundle of fatty tissue obscured the definition of the prostatic apex. This was believed to be the source for the positive surgical margins at the apex [32].

(b) *New Technique. *After reviewing margin data and appropriate video clips of their initial 50 patients, UC Irvine altered their technique [31]. The new technique moved to precisely define the prostatic apex. The method entails 3 steps. First, all fat overlying the puboprostatic ligaments, the DVC, and the anterior aspect of the prostate is removed. The superficial branch of the DVC is essentially always present and divided. Utilizing the 10x stereoscopic vision of the robotic system, we carefully dissect all of the fat completely exposing the entire anterior surface of the dorsal venous complex. The fat is dissected up to the bladder and submitted to pathology for examination. It has been our experience that the occasional anterior prostate positive surgical margins are factitious; sending the anterior fat pad will essentially always be negative for cancer and allows the pathologist to indicate that this margin is negative. Additionally, Finley et al. reported that about 15% of men will have lymph nodes in the fat and on occasion is the only site of metastasis [33]. The endopelvic fascia is incised and the prostate mobilized to the membranous urethra. The second alteration is division of the puboprostatic ligaments and dissection of the levator fibers adherent to the dorsal vein. Again, the robotic system uniquely allows the surgeon to visually differentiate the muscle from the wall of the vein, plus the machine precision allows detailed dissection without injuring the vein avoiding problematic bleeding. This increases the length of exposed DVC which facilitates its stapling. The last step replaces the step of suture ligation of the DVC with the one-step stapling and division of the DVC using a 45 mm endo-GIA stapler (Ethicon, Somerville, NJ). An 18 French Foley helps to identify the urethra. It is important to wait 30–60 seconds before firing the stapler as this compresses the edema from the tissue creating a more secure staple line. Once the stapler is fired, the staple lines converge on top of the urethra in a V configuration. After the prostate has been freed, the thick fibromuscular tissue surrounding the urethra is divided using the point of the V as the reference starting point [25].

**Figure 3:**
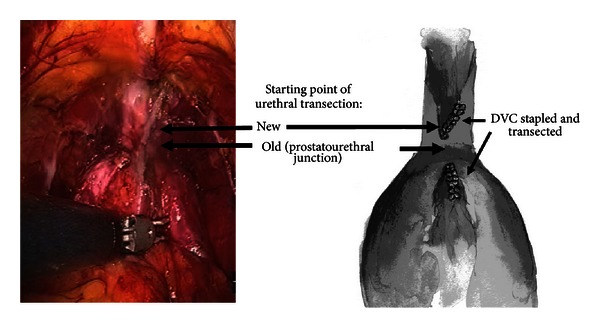
Borin et al. described a more aggressive urethral resection resulted in marked reduction in overall Positive Surgical Margins without significantly affecting time to overall continence [31].

Ahlering and associates continued to refine meticulous apical dissections in an attempt to further reduce PSMs at the apex. Borin et al. [31] showed that a more aggressive urethral resection resulted in marked reduction in overall PSMs without significantly affecting time to overall continence (Figure 3). Evaluation of 200 single surgeon consecutive cases (group 1) revealed that 75% of PSMs occurred at the apex. Assessment of visual cues for urethral length demonstrated that patients with very short urethral stumps requiring perineal pressure during the vesicourethral anastomosis, had equivalent time to continence and overall continence rates compared to patients with readily accessible long urethral stumps. Consequently, the point of urethral transection was altered to include 3–6 mm more of urethra. Time to continence and PSMs for the ensuing 100 cases (group 2) was prospectively followed to evaluate this technical modification. The overall PSM rate for group 1 was 17.6% versus 6% for group 2. In group 2, both pT2 and pT3/4 PSMs were further reduced with this new surgical approach (7.3% versus 2.4% and 50% versus 26.7%, resp.). Kaplan-Meier time-to-continence curves were not significantly different at 3 and 6 months with continence rates of 73% and 89% in the early group versus 61% and 95% for new transection group.

#### 2.5.2. Bladder Neck Transection

The bladder neck transection can be a problematic for novice surgeons, due to the junction's innate natural anatomic variability and the absence of obvious visual landmarks [21, 25]. The assistant plays a critical role identifying the junction between the bladder neck and the prostate [19]. Switching the camera to the 30-degree down scope, first identify the posterior-lateral contour of the prostate. A locking grasper placed via the 12 mm nondominant port bunches and grasps the 12 o'clock adipose tissue of the anterior bladder approximately 2 cms cranial to the prostate. This serves to expose the lateral contours of the prostate suggesting a starting point for the dissection of the prostatovesical junction. The anterior bladder is dissected away from the prostate and the bladder neck is entered. The balloon is deflated and secured with a locking grasper (4th arm or assistant) through the eyelet of the catheter. The external outlet of the catheter is clamped to prevent CO_2_ from leaking. The catheter is pulled externally and secured to the drapes with modest tension. After transection of the posterior bladder neck, the vas deferens and seminal vesicles are identified and fully dissected. 

#### 2.5.3. Rectum and Cautery Free NVB Dissection

After the seminal vesicles are dissected, they are grasped with the locking grasper and pulled anterior and cranial. Denonvilliers' fascia is entered in the midline, and the rectum is mobilized to the level of the apex of the prostate. This delineates the prostatic vascular pedicles. Laparoscopic bulldog clamps (30 mm, shown at left) may be placed on the vascular pedicles at least one centimeter from the prostate. Alternatively, Hemolock clips may be used or the artery can be transected and then judiciously cauterized with a brief burst of monopolar or bipolar energy. Once the vascular pedicles are divided, only scissors are used to complete the NVB dissection. The lateral prostatic fascia is incised high along the prostate, and the NVB is gently dissected off of the prostatic capsule. After completely mobilizing the neurovascular bundle down to the urethra, the urethra is divided sharply. The prostate is removed and the NVBs are observed for small arterial bleeders that are controlled with precise placement of 4-0 suture ligatures of absorbable material. The vascular pedicles are also controlled with the precise placement of 3-0 absorbable sutures as needed. 

#### 2.5.4. Release of the Neurovascular Bundle

**Figure 4:**
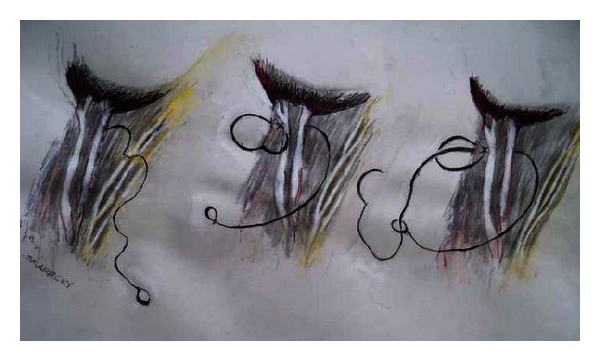
Transition stitch for neurovascular bundle. A distal loop is first created. The pedicle stitch is thrown through the vascular pedicle in a figure of eight fashion then passed through the distal loop to create a tourniquet around the pedicle [30].

With the posterior dissection complete, the lateral prostatic vascular pedicles are well exposed as pillar-like structures. There is general agreement that once the vascular pedicles are ligated, an athermal technique should be used to complete the dissection of the NVB off the prostate [21]. Ahlering and others [35] have shown that athermal technique is essential in preventing local heat injury to the nearby NVB, via the use of locking hemostatic clips or bulldog clamps [36]. We modified our initial bulldog clamp technique and now employ lasso-style adjustable stitch of the vascular pedicles (Figure 4), avoiding “windows” in the pedicle for placement of clips, which often leads to bleeding. It also simplifies the accurate placement and removal of clamps [30]. 

#### 2.5.5. Urethral Anastomosis

In one's first few cases, the bladder neck can be difficult to identify, but as experience grows this problem wanes [21]. Groups now may prefer to stabilize the posterior anastomosis with a “Rocco” stitch [37]. In our experience, this makes the anastomosis much easier, dramatically reduces postoperative hematuria, and might improve time to continence. Then, we use a slightly modified “one knot” suture (van Velthoven stitch) [34]. The running suture is prepared by tying together the ends of two 6 to 7-inch sutures of 3-0 polyglycolic acid, one dyed and one not dyed for identification purposes. Perineal pressure if needed is applied during the initial throws of the suture. The running stitch is initiated at the 4 o'clock position to the bladder, and after 5 throws, the bladder is cinched down to the urethra; it is then run clockwise to the 10 o'clock position. The second un-dyed suture is run counter-clockwise to the 10 o'clock position and the two are ligated.

**Figure 5:**
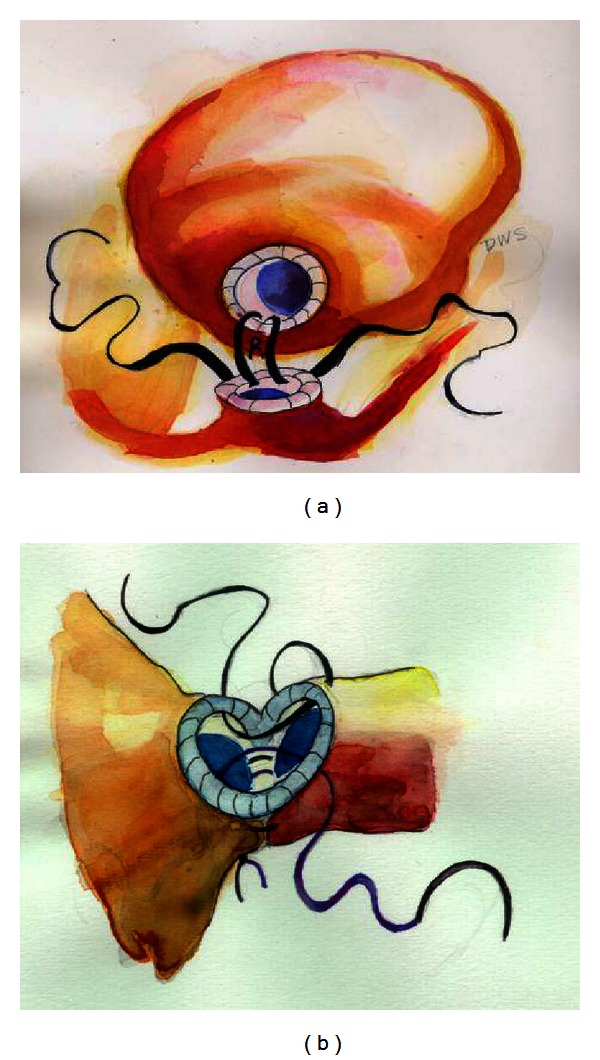
The single knot anastomosis “van Velthoven stitch” [34].

The standard van Velthoven stitch is accomplished as follows: starting with an SH or UR-5 outside-in through the bladder neck and inside-out on the urethra, one at 5:30 and the other needle at 6:30 o'clock (Figure 5(a)). The sutures are run from the 6:30 and 5:30 positions towards the 9:00 and 3:00 o'clock positions, respectively. The posterior lip of the bladder neck is left (1-2 cm) apart from the posterior urethra as the first two throws on the urethra and the first three throws on the bladder are completed. When this is achieved, gentle traction is exerted on each thread simultaneously or alternately; the system of loops acts as a “winch” to bring the bladder in contact with the urethra without excessive traction. A transition suture is completed on either side at 9:00 and 3:00 o'clock, by taking an extra bite on the bladder, going inside out (Figure 5(b)). At this point, an 18 French sialastic catheter is placed into the bladder. Carrying the suturing up to the 12 o'clock position on both sides, going outside-in on the urethra and inside-out on the bladder completes the remaining closure. At 12:00 o'clock, the ends of the running sutures are tied to one another on the outside of the bladder. As such, both knots reside on the outer bladder side of the anastomosis. If discrepancy persists between the diameters of the urethra and the bladder neck, the remaining anterior opening of the bladder is closed in two layers with the same sutures. The balloon on the 18 French sialastic catheter is filled with 10 ccs of water; the bladder is irrigated until clear with approximately 60 ccs of sterile water [25, 34]. 

Prior to removing the prostate and undocking the robot, a final hemostatic check of the entire surgical field must be performed. The prostate specimen is removed through the umbilical port, which affords the most space, although enlargement of the umbilical incision may be necessary for larger prostates. The fascia is then closed with a looped 0 absorbable suture such as PDS (Ethicon, West Somerville, NJ), in a transverse direction, which reduces subsequent hernias. Although many groups do not routinely place a surgical drain at the end of the procedure, [21], it still remains a surgeon's discretion especially if there is a risk of bleeding. Skin incisions may be closed with absorbable subcuticular sutures, skin staples, and/or a biological adhesives.

#### 2.5.6. Pelvic Lymph Node Dissection

A pelvic lymph node dissection is carried out if indicated. It normally prolongs surgery by 15–30 minutes per side. We urge that an extended lymph node dissection should be done including all 3 regions as described by Studer. It is important to obtain a total lymph node count of 10–20 nodes for a complete bilateral dissection. It is a surgeon preference to leave a drain or not.

#### 2.5.7. Removal of the Prostate

Removal of the prostate through the midline incision is simple, free of bleeding, and the easiest port site to close. The entrapment bag string is transferred from the assistant's 12 mm port to the robotic 12 mm camera port. The remaining robot arms are undocked and the robot is pulled out of the operative field. A drain may be placed at this point using the da Vinci camera for guidance. The camera port incision is extended laterally using the port to lift the abdominal fascia and carefully incise the fascia. The port is removed and the specimen bag is pulled gently in large circular motion to extract the bag with the smallest possible incision. The fascia is closed transversely with a looped 0-prolene.

### 2.6. Perioperative Patient Care Issues (Box 2)

 In order to ensure both safety and efficiency in the operating room, patient positioning should be a process that involves every member of the surgical team. Anesthesia should be made aware of the steep Trendelenburg positioning that will be required and aware problems of maintaining a patient in this position for long periods of time. It is highly recommended that a dedicated Anesthesiologist should be a committed member of the robotic team, to insure long-term success of any hospital program. 

 The patient is initially placed in the supine position, with arms tucked and padded at their side. The use of shoulder rests should be avoided as this can lead to serious brachial plexus injury. The legs can either be placed on spreader bars and gently separated or placed in padded boot stirrups in a low lithotomy position. The primary complication to avoid is hyperextension of the femoral nerve when using spreader bars and careful padding of the posterior knee to prevent nerve impingement when using stirrups [25]. 

 Postoperative pain management should minimize narcotics as much as possible. Unless contraindicated, patients are started on ketorolac during port closure, but always prior to extubation. The goal is to have the patient pain free as they awaken in the recovery room. Patients are routinely discharged from the hospital on postoperative day (POD) 1 with prescriptions for ibuprofen and acetaminophen if needed. Avoidance of narcotics reduces postoperative constipation of prolonged bloating. 

 As part of routine antithromboembolic care, thigh-high external pneumatic compression is used both during surgery and postoperative period. Early ambulation is suggested the evening of surgery, and patients are encouraged to walk as much as one half mile on POD 1.

 Urethral catheters are routinely removed on POD 7. Cystography is reserved for the few cases of hematuria or if a complicated BN reconstruction was required. 

#### 2.6.1. Sexual Function

The mainstay to sexual function preservation is avoiding nerve transection followed very closely by reduction of traction and thermal injury. Theoretically, with excellent surgical field visibility due to 10X magnification and decreased blood loss, nerve preservation should be very feasible with the da Vinci surgical system.

Techniques such as bi-polar electrocautery, harmonic scalpel, and ligasure have been introduced in an attempt to reduce thermal and stray electrical injury to the neurovascular bundles. However, in a dog model, Ong et al. [38] demonstrated significant decreases in erectile response when using monopolar and bipolar hemostatic cautery in close proximity to the NVBs. Ahlering et al. [35] previously described their cautery-free, clip-free dissection of the cavernous nerves to decrease nerve injury during RALP and, hence, improve sexual function. Their current technique involves placing bulldog clamps on the lateral pedicles prior to cautery-free, sharp dissection of the pedicles and the NVBs off the prostate. Beck et al. [39] proposed using a pedicle stitch which is a 6 cm 3-0 monocryl on an SH needle with a small loop tied in the suture end (Figure 4). The pedicle stitch is thrown through the vascular pedicle in a figure of eight fashion then passed through the distal loop to create a tourniquet around the pedicle. A small Hemolock Clip is used to cinch down on the NVB to achieve cautery free transaction of the NVB.

In summary, regardless of the specific surgical technique used to preserve the NVBs, to help maintain sexual function, a cautery free technique (clips, bulldogs, etc.) or “minimal cautery” at the vascular pedicle and minimizing traction of the nerves is recommended. 

## 3. Postoperative Complications

 The safety of the robotic approach was initially compared to the laparoscopic or open procedures and is essential for the continuance of the robotic approach [40, 41]. The overall complication rates of robotic prostatectomy have been reported to be between 2.3% and 18% depending on surgeon experience and number of cases in contemporary analysis [42–46]. One drawback is the limited literature on methods of prevention of complications in RARP [21, 47, 48] and their effectiveness beyond just gaining greater surgical experience. 

### 3.1. Early and Late Complications

 The overall reported complication rates among RARP surgeons include minor complications (Clavien 1 + 2) from 5% to 7% and major complications (Clavien 3 − 5) about 4% [21, 49]. Unfortunately, standardization and underreporting complications remain a concern. Reductions in transfusion rates by LP and RARP have primarily decreased the rate of low risk Clavien 2 complications, but they can overwhelm comparison studies to open surgery to statistically favor RARP. Although the transfusion rates are significantly reduced for RARP and laparoscopic RP, one must take into account *all complications*, particularly major complications of all RP surgeries for a valid comparison. For RARP, mortality rates are consistently rare (0.1%–0.2%) [25, 49]. Three mechanisms can assist the surgeon to the correction of complications: (1) the time honored "learning curve," (2) simple passive observation of one's complication rate, and (3) active correction of significant problems.

Rectal injuries most commonly occur during the dissection of the prostatic apex, if not completely mobilized off of the posterior aspect of the prostate. The rectum that remains adherent to the apex is at risk of injury during transecting of the urethra, or less commonly occur during the posterior dissection. To stay in the correct plane, the perirectal fat must be used as a guide, dissecting near the prostatic surface. A second caveat is avoiding lateral rectal injuries during wide resection of the NVBs for cancer invasion particularly at the apex, when performing nonnerve sparing RARP [49]. 

### 3.2. Prevention of Complications

 While the passive learning curve is considered the most important and perhaps the sole method to reduce complications, surgeons occasionally actively modify techniques, if they note a series of complications having a negative impact on the patient. Use of meticulous of followup and documentation can identify specific complications where a technical “fix” can be applied. For example, major side effects (urinary incontinence, erectile dysfunction) and complications (intraoperative bleeding) had occurred during the early open retropubic radical prostatectomy. Addressing these problems by anatomic investigations and innovative surgical techniques, Dr. Walsh developed the more anatomic approach for radical prostatectomy revolutionizing the outcomes [50]. It is important for novice surgeons to seek guidance from experienced RARP surgeons to diminish their complication rates by shortening the learning curve. 

In a study by Liss et al., [49] combined intraoperative and early complications were 0.8% high risk (≥Clavien 3) and 2.3% low risk (≤Clavien 2) for the 1,000 cases experience. In this study, half of the intraoperative/early and 25% of the late complications occurred in the first 200 RARP cases. These observations invoked deliberate changes in technique to decrease side effects and complications not simply related to the learning curve [51, 52]. Long-term data collection of patient demographics and outcomes was essential to recognizing recurrent complications and ultimately provide deliberative corrective solutions. 

 Liss et al. applied this active approach, and Table 1 represents discrete technical changes instituted abruptly to correct a wide range of Clavien complications [49]. Corneal abrasions were prevented by simply utilizing foam based safety goggles (SunMediGuard 9-0210-00, $5 US dollars) perioperatively. Corneal abrasions may cause significant postoperative discomfort and thought to occur secondary to positional eye edema from the steep Trendelenburg positioning. The foam-based safety goggles should be placed over the patient's eyes for surgery and about 90 minutes in the recovery room, until the patient is fully alert and oriented enough not to rub their eyes. The use of goggles was statistically significant in reducing in this complication and is clinically useful.

*Fossa Strictures *By using a 24 French catheter to protect the urethra during stapling, Yee et al. unknowingly created fossa strictures. They found that catheters 22 French or greater can be associated with nearly a 10% risk of fossa navicularis strictures [54]. They consequently reduced the catheter size and eliminated the stricture rate from 9/131 (6.9%) to 1/693 (0.1%); *P* value < 0.001. It is still surprising that the larger bore has such a significant effect. Thus, only a 16 or 18 French (Fr) Foley catheter should be inserted after the sterile drapes have been placed and fossa strictures will be avoided. This contradicts the recommendation of bladder neck closure by a 22 to 24F catheter by Kundu et al., in their open RP study using a non-van Velthoven anastomosis [55].

#### 3.2.1. Reduction of Incision Hernias

**Figure 6:**
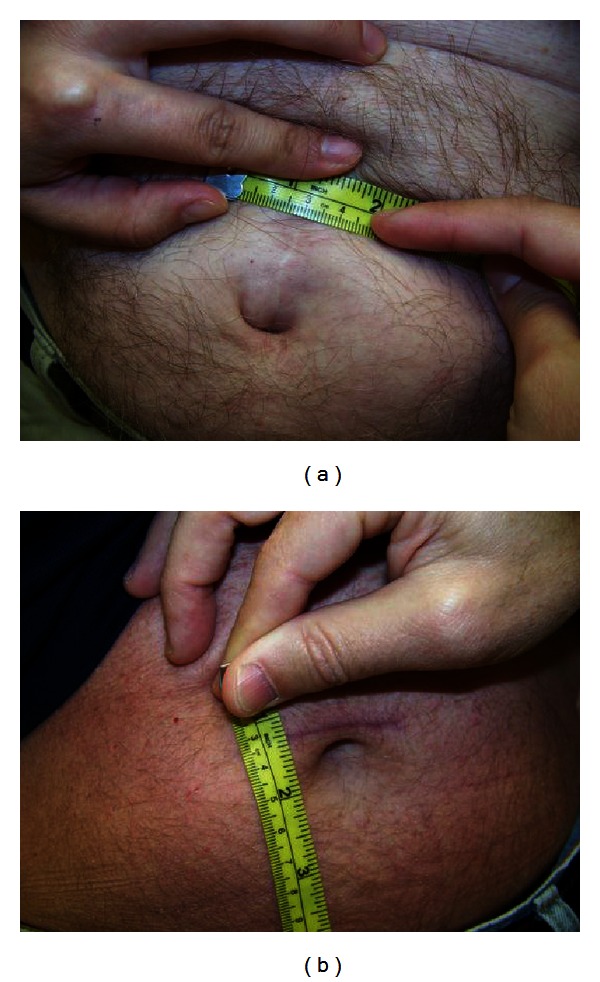
Representative measurements of mid-line (a) and transverse incisions (b) after RARP [30].

The general surgery literature suggested that increased tension may be placed on the vertical fascial incision and lead to questioning the use of the commonly used midline incision RARP to a horizontal fascial incision [56, 57]. Beck et al. presented findings for changing the incision for the camera port from a vertical to horizontal incision [30]. The transverse fascial incision reduced the hernia rate from 4.9% (36/735) to 0.6% (1/165). Figures 6(a) and 6(b) demonstrate the resulting smaller scar. The result is guarded due to lag time bias as many (50%) of incisional hernias present more than 1 year after surgery. 

 Passive observation can be made if a reduced complication is noted from a change made with different intentions. The addition of the Rocco stitch, which reapproximates Denonvilliers fascia prior to the van Velthoven anastomosis, may have led to a subsequent reduction of bladder neck contractures by relieving tension on the anastomosis. Liss et al. did see a trend in reduction of bladder neck contractures [49]. In the first 600 cases, five bladder neck contractures (<1%) occurred and since incorporating the Rocco stitch 400 cases ago, there has been only one bladder neck contracture. 

Identification of complications and proposing insightful working solutions has decreased their incidence in robotic prostatectomy in both major and minor complications. Inclusion of these techniques may significantly improve patient outcomes for robotic surgeons in their early experience.

## 4. Reducing Positive Surgical Margins after RARP

The gold standard treatment for localized disease has long been radical prostatectomy, due to advantages of precise staging and grading and the opportunity of disease abolition, provided there is early detection prior to metastasis. It is a tribute to the landmark anatomical dissection described by Walsh, that in the succeeding 30 years of RP, the morbidity has declined, as functional and oncological outcomes have greatly improved notably over the last decades [22, 58]. 

 Clearly, the primary goal of radical prostatectomy remains cancer control or eradication for patient survival. Prediction of RP long-term oncological outcomes is linked with many established clinical factors: preoperative prostate specific antigen (PSA), clinical and pathologic staging, Gleason grade, seminal vesicle invasion (SVI), lymph node invasion (LNI), and positive surgical margins (+SM). Of this sphere of factors, surgical technique can impact but only one of adverse prognostic predictors, that is, surgical margins. If the cancer is organ confined (pT2), +SM is likely attributed to surgeon error, incorrectly resecting the prostate during RP.

A positive margin after radical prostatectomy (RP) suggests surgeon error, many vital organs surrounding the prostate confront the surgeon, that is, rectum, pelvic sidewall, bladder neck, and urogenital diaphragm, which complicate a complete and clean resection. It is widely supported in the literature that +SM predicts adverse oncological outcome (PSA recurrence), although there are dissenters [59–61]. The five-year biochemical recurrence rate (BCR) in men with +SM ranges from 42% to 64%. Reduction of +SM rated has been demonstrated by modifying surgical techniques, Figure 3 [31, 32]. Optimal treatment of men with +SM remains controversial as to when to employ adjuvant treatments; so, the quest for surgeons to totally eliminate +SM is an attractive but elusive goal.

### 4.1. Do Surgical Margins Matter?

The long-term impact of +SM compared to other clinical factors such as stage and Gleason score remains contentious. Many agree it is an independent poor prognostic indicator resulting in higher biochemical recurrence [59, 62–65]. The reported five-year biochemical failure risk for +SM is between 42% and 64%, significantly increased over men with negative surgical margins (−SM) [64]. Swindle et al. reported on nearly 1,400 RRPs [59] and found that overall the +SM rate was 12.9%, with 6.8% for T2 and 23% for T3 clinical stage and 10-year BCR-free rates of 81% (±3%) for −SM and 58% (±12%) for +SM cases. They rendered a relative failure risk of 1.2–2.7 with +SM, even after adjusting for concurrent risk factors (pretreatment PSA, Gleason grade, and clinical and pathologic stage). Lee et al. demonstrated similar findings in another large study of 2,500 RRPs [25]. 

It is intuitive that residual cancer of a +SM impacts BCR in pT2 tumors, but is this impact diminished if the cancer has already escaped the prostate, exploiting routes via the capsule, seminal vesicles, or lymph nodes (stages pT3a, pT3b, pT4)? Karakiewicz et al. reviewed the impact of +SM on 5,831 patients in a multi-institutional study reporting +SM increased the BCR risk 3.7-fold enduring through 10 years [66]. Positive surgical margins increased the likelihood of BCR even in men with extracapsular extension and positive seminal vesicles (SVs), but interestingly not in cases of positive lymph nodes. 

### 4.2. Positive Surgical Margins

The positive margin rates from large contemporary RARP series range from 9% to 29% [22]. There are several caveats to note when interpreting surgical margin rates between series. First, there is not standardized reporting between institutions. A second cautionary note if comparing overall +SM rates is that many times they are reported as a combination of pT2 and pT3 staging. However, the ratios of pT2 versus pT3 rates vary between referral and nonreferral centers, and stage pT3 +SM rates are higher due to greater volumes of cancer and Gleason scores. Thus, institutions with greater percentages of patients presenting with pT3 tumors will naturally have skewed higher overall +SM rates. 

#### 4.2.1. New Techniques

Beyond technology advances and the surgical learning curve, technical refinements have proposed to reduce positive margins. Ahlering et al. [32] described a method to decrease pT2 margins, which improved their overall +SM rate from 36% to 16.7% and from 27% to 4.7% in pT2 cases. They compared their initial 50 cases to their next 200 consecutive cases and suggested three technical steps to aid in the apical dissection: (1) removal of all fat overlying the dorsal venous complex (DVC) and prostate, (2) full dissection of the levator fibers to expose and increase the DVC length, division of the puboprostatic ligaments, and (3) division of the DVC using a laparoscopic vascular stapler. 

Borin et al. described a second modification to the apical dissection, which further reduced +SM [31] primarily in pT3 stages. They noted that in their previous cases, 75% of +SM were focal and located at the apex. They altered their point of urethral transection to include 3–6 mm more of urethra, and the overall +SM rate declined from 17.6% to 7.5% with the modified technique. Importantly for pT3/pT4 staged tumors +SM rate declined from 50% to 13.8%. These technical modifications should be validated by other institutions, to establish that these techniques are translatable, and not solely surgeon dependent. 

## 5. Robot-Assisted Radical Prostatectomy: Oncologic and Biochemical Outcomes

Oncologic cure is the primary intent of RARP. The early widespread adoption of RARP without supporting long-term recurrence or survival data rightfully invoked concern among the radical prostatectomy community. These early fears are allayed in that recent intermediate-term oncologic results suggest comparable outcomes to standard open RRP. The oncologic data with traditional RRP is more substantial, but as shown by several groups between the United States and Europe, RARP is establishing its merits within large institutions as a valid option in the surgical management paradigm of localized prostate cancer [22].

 Since its introduction, there have been limited studies describing biochemical recurrence rates in men undergoing RARP as this is a fairly new technology applied to a relatively prolonged disease course [67]. It is understood that the prostate specific antigen serum test can provide monitoring for cancer recurrence after prostatectomy [67]. In an extensive review of open radical prostatectomy, the 5-year biochemical recurrence rate was about 25% (16%–31%) [68].

### 5.1. Year Biochemical (PSA) Recurrence Findings for RARP

 Robotic-assisted radical prostatectomy was largely introduced in 2001-2002 for the treatment of localized prostate cancer and has become widely disseminated in the United States. Now with experience nearing 10 years, the opportunity to report clinically relevant 5–10-year followup is now accessible from four institutions, two each from the United States and Europe. 

 It must be acknowledged that Mani Menon and his colleagues at Henry Ford Hospital largely pioneered RARP in the United States, and in 2010, Menon et al. were the first team to report on RARP patients having a median of 5-year followup (median 60.2 months) [69]. The 5-year BCR-free survival was reported at 87% in 1,384 patients. A Swedish group led by Peter Wiklund, another early RARP pioneer, has also reported on 5-year outcomes on 944 men, median followup of 6.3 years [70]. Their RARP study was strengthened by the comprehensive availability of followup PSA results via the Swedish Registries. The overall biochemical recurrence free rates were 84.8%, and 87.1% at five and 84.5% at seven years. The second European group to report on 5-year BCR-free survival in a smaller group of 184 men was Suardi and colleagues in 2011. With a median followup of 67.5 months they reported an overall 5-year BCR-free survival of 86% [71]. Liss et al. reported the Ahlering, UC Irvine series, of 433 consecutive patients, with comparable baseline oncologic characteristics [53]. The cohort represented a loss to followup of only 1%, while 63% (*n* = 272) had ≥5 years followup and 88% (*n* = 391) had ≥3 years followup and found BCRFS of 86%. 

 In the 4th study, Liss et al. included adjuvant therapy patients in their analyses and counted these patients as having a BCR. Also, if adjuvant radiation therapy was done for PSM, those patients were considered to be a BCR, as to not underestimate the number of BCR. Overall BCR-free survival in this series was 84%, considering all of the discussed. The series also had low prostate cancer specific mortality with only 4 patients succumbing to the disease in this 5-year cohort. 

 It is remarkably that all four long-term studies give nearly identical results, especially since these studies represent the early learning curve for all four groups and different geographical populations. These results also compare favorably to the open radical prostatectomy data with BCRFS ranging from 78% to 92% [22]. 

 The most important factors for BCR progression are high Gleason grade and pathologic stage pT3b or seminal vesicle involvement (Table 2) [53, 70]. Another predictor of BCR is positive surgical margins (PSMs), but at reduced hazard ratios. Positive surgical margins in RARP are similar to previous open and laparoscopic series as well [22]. As with any new technology, comparison to the standard operative technique is imperative to justify the continuance of the new technology. In a recent multi-institutional study of robotic prostatectomy PSM with over 8,000 patients, the overall rate of PSM was 15.7% [72].

## 6. Preserving Continence

 After cancer control, urinary incontinence and its sequelae are the greatest concerns for men after RARP. In a large scale international review of incontinence after RARP, Ficarra et al. reported one year pad free continence rates ranged from 69% to 96% (median 84%) and 89% to 92% if a relaxed definition of no pad or security pad is defined as incontinence [23]. The combined authors noted urinary incontinence predictors of age, BMI, comorbidities, LUTS, and prostate volume after RARP [73, 74]. In the decade of experience, groups have begun to detail the quality of life of long-term continence/incontinence after RARP and explore methodologies to improve outcomes.

### 6.1. Pad Free Definition of Post Prostatectomy Incontinence

Traditionally, continence definitions within RP studies vary greatly, from pad free continence, or a much broader view of continence which includes “security” or single pad users, and, hence, a greater overall success rate. Thus, potentially each institution could present 3 levels of success, and careful reading of the Methods section is mandatory to understand reporting. However, an important contribution of RARP is “raising the bar” by emphasizing continence definitions as the need for zero pads. Liss et al. showed a clear distinction in satisfaction measured by the urinary quality of life (Bother score) in men following RARP based on how many pads they wear (0, security, 1, 2, 3+) [75]. For men requiring no pads, the mean urinary QOL was rated 1 (pleased), whereas men wearing either a security pad or one pad had a mean QOL of about 3 (mixed). Clinically speaking, men did not see a relevant clinically difference between a security pad and 1 pad, somewhat equally unhappy. 

**Figure 7:**
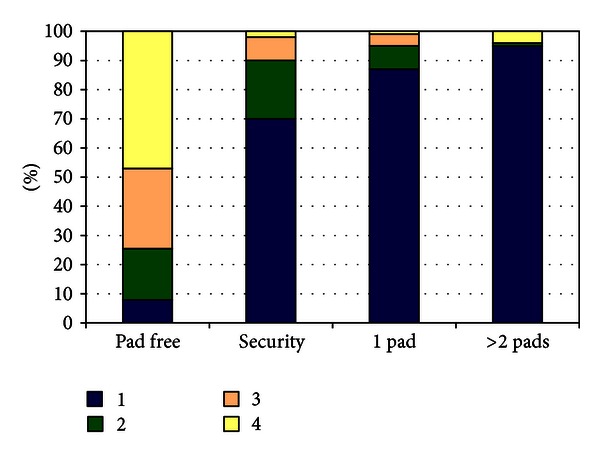
Urinary control after RARP, comparing pad free men versus men who use pads [73, 75]. Scale is 1 = no control, 2 = frequent dribbling, 3 = occasional dribbling, and 4 = total control.

**Figure 8:**
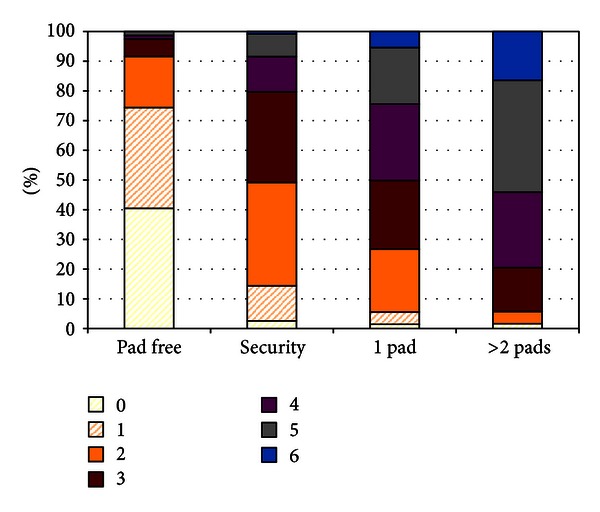
Urinary Bother scores for pad free versus men who use urinary pads after RARP [73, 75]. Bother scale is 0 = delighted, 1 = pleased, 2 = mostly satisfied, 3 = mixed, 4 = mostly dissatisfied, 5 = unhappy, and 6 = terrible.

Figure 7 depicts that pad free men have 75% rate of total urinary control or occasional drippage, while the rates were dramatically less, leaving >90% of pad users with frequent dribbling or no urinary control. The results are similar for Bother score in Figure 8: ~75% of pad free men delighted or pleased versus ≤15% for any pad user. Fifty percent of security and 75% of single pad users had mixed to terrible bother scores. The caveat is that studies of continence which includes security or single pad use include men with frequent leakage and negative bother scores and pad free versus pad usage where the continence is not equivalent.

#### 6.1.1. Age and Influence on Continence

Aside from these technical issues is the impact that patient related factors have on the time to recovery and overall of 0-pad continence. Pick et al. examined the roughly 10% of men who were incontinent (i.e., using any pads), the factor that was immediately obvious was age [76]. In our experience, men over the age of 75 have a 30%–40% rate of requiring pads long-term compared to ~10% for 70–75, ~3% for 65–69. Technically, surgeons do not perform a significantly worse or better operation for a 76-, 66-, 56-, or 46-year-old person, but clearly the younger the man, the quicker and better the continence rates. Pick found that technical factors: learning curve, Rocco stitch, nerve sparing status, and thermal versus athermal transection of the apex and bladder neck, did not significantly impact outcomes. However, patient-related factors included age, Bother score, IIEF-5 score, BMI, and particularly learning curve, medical comorbidities (individual and accumulated). The findings are intuitive as they relate to vitality [77].

#### 6.1.2. Achieving Continence without Sacrificing Positive Surgical Margins

Another technical factor that did not appear to influence time to continence or overall continence was the point of transecting the apical urethra. Although Borin et al. reported a lower point of transection on the urethra to reduce anterior apical surgical margins and they saw no impact on continence, but positive surgical margins were reduced to 7.5% [31]. That study found that the urethral length taken had only a marginal effect on early continence, and that moving the cut 3–6 mm distal to the prostate had a significant reduction in +SM tpT2 and pT3 rates. It should be noted that the cancer rarely exudes apically into the inner membranous sheath, almost exclusively invading the outer muscle sheath. 

#### 6.1.3. Is Nerve Sparing Necessary for Continence—A Long Running Controversy

 A relationship may exist between return to continence and preservation of the neurovascular bundles (NVBs) for potency. Problems of low numbers of non nerve sparing (NNS) men, nonmultivariate analyses without inclusion of IIEF-5, age, and BMI have brought controversy to this issue. Recent reports evaluated associations between baseline characteristics, nerve sparing status and return of continence [76, 77] and did not found a convincing relationship. Tzou et al. [78] presented an insightful finding stating that “men undergoing nerve sparing surgery, with or without return of sexual function, had no better return of continence then men undergoing nonnerve sparing surgery.” 

### 6.2. Avoiding Continence Impairing Complications

#### 6.2.1. Bladder Neck Contractures

In general, continence outcomes after RARP have been good, with reported 85%–90% pad free rates for men [23]. What has positively influenced continence outcomes is the early introduction of the van Velthoven stitch (single knot anastomosis) that profoundly impacted continence following LRP and RARP [34]. With the van Velthoven stitch surgeons can achieve, with nearly 100% confidence, a watertight anastomosis. The single knot stitch originally described by van Velthoven, allowed for the creation of a running urethral anastomosis a tension free fashion. The critical benefit of this technique is that the initial tension of approximating the bladder to the urethra is dispersed over ten needle holes rather than two with interrupted techniques. It is simple and creates a watertight anastomosis with only one intracorporeal knot required. We applied the van Velthoven from case 1 onward, and the BNC rate for all men was originally <1%. In a combined experience of more than 2,000 cases, the bladder neck contracture rate as reported by our group at UCI and groups at the University of Pennsylvania and Ohio State was <1% [79].

#### 6.2.2. Rocco Stitch Postoperative Urinary Retention/Clotting

A second improvement to the reconstruction of the bladder to the urethra is the Rocco stitch, first described in 2006 [37]. Debate exists as to whether it improves time to continence. Although a number of authors have reported that the Rocco posterior suspension suture has improved continence, our experience and that of Menon and associates is that it does not impact time to continence [80]. However, we and others strongly recommend incorporating the Rocco stitch as it further facilitates a tension free anastomosis. It has markedly improved the ease of performing the van Velthoven stitch by reducing tension. It is also very hemostatic, as it is rare now for patients to have visible hematuria at the time of discharge on post operative day. It has nearly eliminated ER and clinic visits for hematuria and clot retention. Another anecdotal finding at this point is the reduction of bladder neck contractures [49]. 

### 6.3. Prediction of Postoperative Urinary Incontinence

The prediction of how much time it will require for any given patient to achieve pad-free status is a strategic clinical goal for early interventional treatments. Although this is extensively studied, there are no reliable preoperative or baseline factors that predictor time to continence [55, 81, 82]. Patient age is the one demographic factor most commonly linked to continence and time to continence; however, the predictive strength of age and all other baseline factors such as BMI, prostate weight, and AUA symptom score are weak at best [83–86]. Additionally, we found that preoperative uroflowmetry (voided volume, PVR, and PFR) had no predictive findings [87]. 

Since preoperative factors have not demonstrated benefit, a number of authors have focused on postoperative factors. In 2000, Twiss et al. [88] were the first group specifically addressing postoperative factors that helped stratify time to continence. More recently, two groups in Europe have published postoperative protocols to predict time to continence. Ates and associates [89] described a urine loss ratio (urine loss ratio: weight of pads/24 voided urine volume) measured on the day of catheter removal or approximately 2.3 days later. They defined three groups of continence, early (3 M), midterm (3–12 M), and late (12–24 M). They found reasonable correlation of being pad free at 3 months when the ULR was 0–.05: 89.4%, .05–.15: 73.5%, and >.15: 42.5%. They commented on better correlation if they measured 2.3 days versus 1 day after catheter removal. Recently, Van Kampen et al. [90] also described a pad-weight method on the day of catheter removal. 

Recently, our group replicated these earlier studies without requiring pad weights and was also performed between 4 and 7 days after catheter removal. Skarecky et al. [87] found that simply filling out a postcatheter log of daily pad-use strongly predicted prolonged urinary incontinence; for men using 3+ pads, the median time to pad free status was 73 days, for 2 pads the median time was reduced nearly in half, 42 days, and 1 pad the median was 35 days. The pad weight methods described by Ates et al. have very similar estimates as our data does [89, 90]. For example, in the Ates study, the 0–.05 ULR correlates well with 1 pad estimates for continence at 3 months, the .05–.15 with 2 pads and >.15 with 3+ pads (89.4% versus 83.6%: 73.5% versus 71.4%: 42.5% versus 52.3%). Another interesting finding was that the first 24 hours period was not the optimal day for predicting time to continence. In fact, 34.3% of men using 3+ pads on day one after catheter removal were using 2 or fewer pads on day 4. All three studies use either the day of catheter removal or 1-2 days later. Another finding was stable pad usage days 4–7; any of these days had equal predictability of time to continence. Previous findings put together introduce a fairly simple means to estimate how long the incontinence may persist. The sum of these 4 publications information can be used both to counsel patients and to direct them to earlier intervention. Early noninvasive intervention techniques such as biofeedback and muscle strengthening should be investigated as well as earlier surgical interventions could be considered.

### 6.4. Future Continence Preservation

#### 6.4.1. Hypothermia

Finley et al. [91–93] hypothesized that a contributing factor to the delay in recovery of post-prostatectomy continence and potency was due to inflammatory reaction secondary to surgical traumatic such as mechanical forces and thermal energy. There is widespread evidence that hypothermia mitigates all inflammatory pathways and improves repair mechanisms. Controlled hypothermia impacts a range of biochemical, histological, and physiological effects which include a temperature dependent reduction in cellular metabolism leading to reduced energy demands [94], decreased free radical production [95], interruption of the apoptotic cascade preventing cellular injury from leading to irreversible apoptosis [96–100], and perhaps most importantly, decreased inflammation by reducing polymorphonuclear leukocyte invasion and chemotaxis [101] as well as reducing proinflammatory cytokine production [102–106].

**Figure 9:**
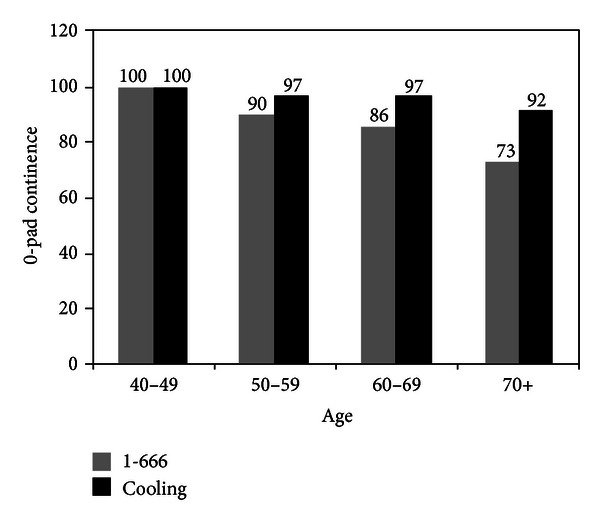
Comparison of pad free continence rates at 12 months after RARP. Gray bars represent noncooled men during surgery [76]. Black bars represent the rates of pad free men who underwent hypothermic cooling during surgery [91, 92].

The UC Irvine group devised a novel technique to apply locoregional hypothermia to the pelvis during robot-assisted radical prostatectomy (RARP) to reduce inflammatory injury [91]. Regional pelvic cooling (<30°C) was achieved with a prototype endorectal cooling balloon (ECB) during the course of RARP. Continence was defined as no pads. Median time to zero pad use was 39 days versus 62 days (hypothermic versus controls *P* = 0.0003). At 1 year overall pad-free continence was 96.3% versus controls of 86.6%; *P* < 0.001. Hypothermia using a preliminary prototype ECB, the time to continence was significantly improved. The impact of hypothermic cooling increases in the older age cohorts (Figure 9), and older men (e.g., ≥70 years) enjoyed the greatest improvement in overall long-term continence [92]. Thus, all men undergo exactly the same procedure (e.g., apical dissection, Rocco plication, and van Velthoven anastomosis) regardless of age; yet as men get older, they do not fully recover from the (trauma of) surgery and, hence, suffer more permanent incontinence. This suggests that hypothermia reduces inflammation resulting in less overall incontinence. Randomized multicentered clinical trials are still needed for validation of this novel technique. 

## 7. Factors Impacting Potency Preservation

Three decades ago, radical prostatectomy was reinvigorated by the landmark study of Walsh and Donker [107] describing the anatomical dissection of the neurovascular bundles (NVBs). It cannot be overstated the profound improvement upon RP to be able to preserve sexual function. This paradigm shift constituted a new perception to seek new technical strategies to improve quality of life for men after RP [73]. To this day, the literature to totally preserve sexual function is still evolving. Alternative scenarios are controversial, either proposing anatomic preservation versus surgical trauma, or inflammation versus patient related factors such as age, medical and psychological conditions, and others. 

### 7.1. Cavernous Neuroanatomy

 The description of the labyrinthine path of the parasympathetic nerves by Walsh and Donker described the pathways where the pelvic plexus intertwined past the seminal vesicles and then along the posterolateral aspect of the prostate, between the true capsule and the lateral prostatic fascia (the supralevator pathway) [107]. Continuation of these nerves led posterior and lateral to the urethra, ultimately piercing the urogenital diaphragm and follow on to the pubic bone (infralevator pathway). At this point they form the delicate neural interconnections at the penile hilum between the cavernous and dorsal nerves [108, 109]. In the quest to improve potency outcomes in RARP, Tewari and associates [110], Tekenaka et al. [111], and Costello et al. [112] have reinvestigated precise gross and histologic dissections of male cadavers, delineating the cranial and caudal paths of the cavernous nerves. 

Menon et al. proposed the “Veil of Aphrodite” technique of preserving the prostatic fascia and reported on 53 men, finding a 97% return of sexual function at 12 months in a comparative study to 23 control men of only 74% potency return [113]. Similar to Menon, Costello concurred that the NVB passages along the posterolateral border of the prostate surrounding the lateral pelvic fascia, the pararectal fascia, and Denonvilliers' fascia. Their findings contrasted with Menon, in that the nerves located within the veil of Aphrodite innervate the prostate and were sympathetic in nature, but also branching to the levator ani and anterior rectum, thus not likely as critical to potency as Menon et al. proposed [113]. That the techniques are still evolving is evident in that the 2012 Pasadena Consensus Panel found wide variability of the techniques used by the panel's surgeons and made no recommendation of a standard approach or surgical technique [21].

 Surgical techniques for return of sexual function after RARP are greatly varied, and the return of potency is promising but vastly problematic for many men postoperatively. Clearly preservation of the nerves is a critical component, as identified by Walsh and Donker [107], but other patient and surgical characteristics may account for the wide variability of “potency outcomes” reported in the literature. The delay in the return of sexual function after RARP provokes two interesting questions: why do some men recover immediately and others at one, two years or longer? Also, what characteristics impact who recovers? 

### 7.2. Definitions of Nerve Injury

**Figure 10:**
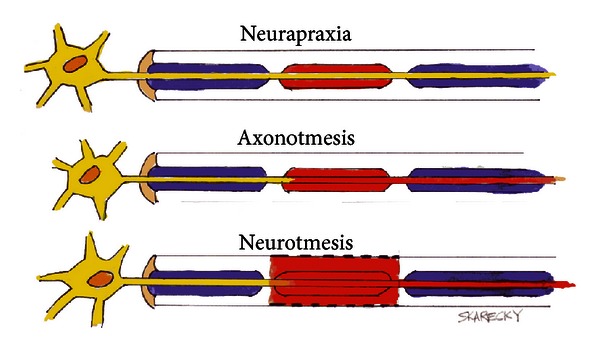
Classification of nerve injury according to Seddon [114, 115].

A physiological avenue to be considered is the established mechanisms of injury to peripheral nerves, as opposed to central or spinal cord injuries. A review of the past literature on peripheral nerve injuries finds that they were classified by Seddon, 70 years ago in 1943 [114, 115]. In his initial and simplified classification of injury, three categories of severity were proposed. *Neurapraxia* is a mild injury due to nerve contusion from blunt impact or stretch injury to the nerve with no structural damage (Figure 10, top). This concussion-like state results in a transient conduction block from which full recovery occurs within days to weeks. A moderately severe injury *Axonotmesis* results in axonal disruption and Wallerian degeneration, but the perineurium is preserved (Figure 10, middle). The nerve or axon has the ability to regenerate or regrow from the point of injury to the end-organ provided the perineurium is intact. Notably, regrowth of the axon advances at in the order of one mm/day to one inch per month and recovery taking 8–24 months. The most difficult nerve injury to overcome is *Neurotmesis, *which occurs after severe injury or a laceration that completely transects the axon and perineurium, providing with no capacity for regrowth of the axon, and usually resulting in aneuroma or scar (Figure 10, bottom). During radical prostatectomy, a constellation of excision, incision, severe stretch, or thermal injuries to the pelvic nerves is possible during radical prostatectomy resulting in multiple types of nerve injury suggested by Seddon.

### 7.3. Recovery of Sexual Function via Prevention of Peripheral Nerve Injury

**Figure 11:**
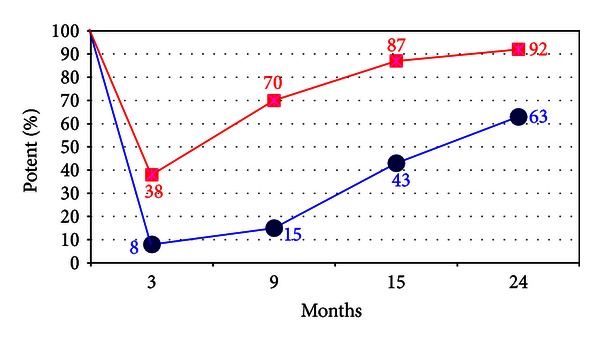
Comparison of improved athermal rates of potency (squares) to rates where cautery was utilized during RARP (circles) [116, 117].

 In their RARP cases UC Irvine experience (cases #1–125) used cautery to fully control the prostatic vascular pedicle (PVP). The circles in Figure 11 (lower curve) show the rate of recovery over the 1st two years [115, 116, 118, 119]. Although the nerves appeared to have been well preserved, potency rates that were only slightly better than anticipated. The use of increased usage of cautery during RARP emerged as a possible basis for depressing the return of potency rates. In response, Ahlering et al. adopted an athermal technique. Thereafter they found a striking increase in potency recovery from 8% to 38% at 3 months [116, 119]. In the cautery group, slow and steady recovery of potency surprisingly also occurred over the two years [117]. An explanation for the 1-2 year delay is that perhaps as some injury to the NVB occurred, the injury was not permanent, and supported by finding that 68% bilateral NVB men recovered by two years, Figure 11. In the athermal group, the results were striking. The elimination of thermal injury improved return of sexual function nearly 5-fold at 3 months, from 8% in controls to nearly 40% in the athermal men, Figure 11 (upper squares). The return of potency via the athermal technique appeared to follow the three mechanisms set forth by Seddon. Transient or no obvious injury (*Neurapraxia*) is likely in men potent in early attempts at intercourse (~40%). A second group of men recovered at around 9–15 months (*Axonotmesis)*, after long delay. The final third group patients were likely to have permanent injury and never recovered at 2 years (*Neurotmesis)*. 

### 7.4. Thermal Injury

 The avocation of athermal technique for nerve sparing during RARP by Ahlering et al. [35] and Gill et al. [36] is not without precedent from other fields. It is well recognized that thermal energy on or near the nerves is major mechanism of damage. Neural injury can be produced by temperature elevations of as little as 4°C (to 41°C) [120, 121]. Coagulation can occur with elevations to 45°–55°C [120], and if temperatures rise beyond that point, cell death occurs, as denaturation occurs at 57°C to 60°C and protein coagulation at 65°C [121]. An important observation by Donzelli et al. is that *both monopolar and bipolar cauteries *cause primarily thermal injury to surrounding neural tissue [122]. 

 The profound impact of thermal injury was presaged in a canine model by Ong et al. in a landmark paper demonstrating the direct effects of electrocautery and thermal injury on cavernous nerves [38]. Ong et al. compared monopolar electrocautery, bipolar electrocautery, and harmonic shears to standard suture ligatures for unilateral NVB dissection. As an internal control, the contralateral bundle was not dissected. After cavernous nerve stimulation, only the energy-free (suture ligature) group maintained similar-to-baseline intracavernosal pressure responses, instantly and two weeks later after dissection. The other methods which relied on thermal energy, resulted in a >95% decrease in the canine cavernosal pressures. These findings were further confirmed via histologic studies demonstrating increased amount of inflammation associated with the use of heat and/or electrocautery. These findings advocated in the animal model that transection of the vascular pedicles should be without thermal energy, unless a simultaneous thermoprotective neutralizing cold irrigation is used. This finding is supported by the 2012 Pasadena Consensus Panel as well [21]. 

 It is perhaps surprising that the damaging effect of electrocautery on the nerve, is not due to mutated electrical stimulation, but the heat generated by the cautery itself. The increase in heat by the electrocautery has been shown to radiate beyond the specific site of delivery, as standard laws of thermodynamic applied to heat dispersion in tissue would imply. In a study by Mandhani et al., the average temperature rise with monopolar and bipolar cautery at the NVB, measured at a more removed anterior black neck >1 cm from the NVB incision, was 43.6°C and 38.8°C, respectively after ~ one minute of cautery [123]. Concurrently, the mean temperatures within the NVB measured within 1 cm of the cautery also rose to 53.6°C and 60.9°C, respectively, using either cautery modalities, and the average time return to precautery temperature baseline with each modality was 3.4 seconds for monopolar and 6.4 seconds for bipolar. It is intriguing that mono- and bipolar electrocautery incur similar temperature increases, but monopolar cautery would appear more efficient in shorter pulses and thus would produce lower overall temperatures. A second study supporting the impact of heat on cautery by Khan et al. [124] confirmed the heat sink effect and its thermodynamic impact on adjacent arteries and veins. They demonstrated in a porcine model, active blood flow through arteries and/or veins consistently dissipated heat. However, if the vessels were clamped, restricting the blood flow, the thermal spread through adjacent muscle mimicked the heat dispersion as if the blood vessels (heat sink) were not present. Zorn et al. have suggested another protective method to mitigate heat damage. They determined that application of cold irrigation applied concomitantly with cautery can measurably reduce the thermal spread [125]. A consensus RARP publication recommends that the simplest solution is to avoid thermal energy altogether near the NVB [21]. Surgeons should be advised to adapt simple thermodynamic principles such as low wattage, short bursts, and distance, to minimize thermal spread. It remains to be studied if a minimum spread of heat with short cautery pulses would fall within the threshold of “safe” electrocautery around the NVB. 

### 7.5. Traction Injury

 Another source of possible nerve injury may be traction or stretch injury [115]. This is a well-recognized nerve injury in animals and perhaps frequent in human complications as well. One example is the urologically familiar femoral nerve stretch injuries induced by prolonged laparoscopic Trendelenburg positioning. Ahlering et al. examined their 3-month potency outcomes in 139 potent men aged ≤65 and determined that although age and prostate weight were significant in univariate analysis, only prostate weight remained significant in multivariate analysis [126, 127]. They also controlled for list of cofactors such as age, BMI, medical comorbidities, medications, social causes, and perioperative factors [126, 127]. They surmised “traction injury” as the cause in that the smallest prostate size had the lowest risk of impotence and increased in a step wise fashion at 3 months continuing over 5 accumulative sized quintiles. That traction injury usually recovers at 9–15 months, explained why prostate size is not a significant factor for recovery at 12 months, in that RARP has already recovered from its impact. These findings were recently supported by Patel et al. (personal communication).

### 7.6. Nerve Redundancy

A long running controversy is the threshold amount of nerves spared during radical prostatectomy to ensure recovery of sexual function. If one excises one of the NVBs, does sexual potency also decline by one half? Is there any evidence that mechanisms exist via “systems redundancy” to enhance single NVB function [115]. Finley et al. compared potency outcomes in RARP for unilateral versus bilateral nerve preservation, with a specific definition of only patients with a wide excision as unilateral [128]. Included in the study was the percent of sexual function recovery following preservation of one versus two nerves (i.e., a doubling of nerve volume) and if differences occurred in the quality of erections. Finley et al. found that if the nerve volume was doubled (2x), there was only a 15% improvement (1.15x) in the cautery-free group and 36% (1.36x) signifying a significant amount of redundancy. Equally interesting was that in men reporting potency recovery, qualitatively the erection in the group with one nerve was the same as in men with two nerves. Average postoperative IIEF-5 scores of both the uni- and bilateral groups were the equivalent, 19.6 versus 18.9 for cautery and 21.0 versus 22.0 in the cautery-free group, respectively. 

 The literature in open radical prostatectomy has similar findings. Walsh et al. [129] found in men potent before RP, 69% undergoing unilateral wide excision returned to potency after RP, versus 85% who had BNS. A similar result was reported by Kundu et al. [55] noting overall potency rates at 18 months, not of 1 : 2 ratio but 53% and 76% after UNS and BNS RP, respectively. Noteworthy of these reports is that if the volume of nerve tissue is doubled, this increases potency rates only by about 1.23–1.43-fold. The existence of redundancy is one hypothesis that explains the weak relationship of quantity of nerves spared to the quality of nerve recovered. 

Overall, care should be taken in RARP to avoid or diminish heat spreading to the NVBs, and minimization of traction injury to preserve sexual function. At the moment RARP may be confounded as to alternate approaches to nerve preservation, as we may have currently exploited the technical preservation of neuroanatomy of the cavernosal nerves. If redundancy is a substantiated mechanism, then the impact of additional “nerve preservation” may be limited in to further improve the return of sexual function in RARP [115]. An alternative path to explore is the prevention of the injury due to inflammation as a potential new direction to improve potency QOL outcomes. Hypothermia or the use of cold to prevent ischemia presents one such novel therapy to help protect the NVBs before, during, and after surgery.

## 8. The Prevention Paradigm 

For younger and healthier men undergoing RARP, they have a greater likelihood of retaining their sexual function after surgery than their older counterparts. Age and health benefits are independent of the surgeon or technique [115, 127]. RARP technique is performed identically as possible across all patients by individual surgeon and does not impart greater trauma on older (70+) than a younger men aged <60. One possible answer may lie in younger men's ability to better respond to surgical trauma and ensuing inflammation. This simple perception reinforces our understanding of age and health, as well as how elements optimize the outcomes in many surgical procedures for the younger men, but reduce recovery in older men. 

Adapting and developing new surgical techniques has been omnipresent in RARP and major reasons for its acceptance in the urological community. Overlooked thus far are the iatrogenic effects the inflammatory cascade may provoke immediately following the trauma of surgery. Surgery induced activation of coagulation factors, proinflammatory cytokine formation, hypoxia, microcirculatory impairment from endothelial damage, acidosis, free radical production, and apoptosis can all lead to damaging effects [91]. Prevention of this cascade by “hypothermia” is evidenced by shielding neurons from damage, averting paraplegia in rabbit studies, and in protective in experimental injury models of the central and peripheral nervous systems [91, 93]. 

In a series of reports, investigators at UC Irvine developed a novel endorectal cooling balloon to apply local hypothermia to prevent the inflammatory cascade within the external urinary sphincter for continence and the neurovascular bundles for sexual function [91–93]. As discussed previously, the hypothermic impact on continence is greater thus far. In very preliminary finding, Finley et al. have also seen some improvement in preserving potency after RARP. When they compared potent men aged 40–78 at their 15 month interval, the recovery rate of sexual function was 83% versus to 66% in similarly aged controls [92]. In harmony with the hypothesis older age may be at risk for inflammatory damage, men over the age of 65 had the largest improvement in potency: hypothermia 70% versus controls 30% [92]. This intriguing finding in the small numbers of men in this study still requires a much larger validation series.

## 9. The “Trifecta”

 RARP in its zeal to demonstrate improve outcomes has proposed a novel measurement, the “Trifecta.” Three elements are combined into the “Trifecta” score: progression free PSA, continence, and potency, and they combined into single score card that counts success when all three are attained [63]. As a “gold standard,” it has several inherent problems. Impotent or men with ED generally are excluded, as well as men not having bilateral nerve sparing, who remain at higher risk of extra prostatic extension and BCR, and this skews Trifecta results. The remaining men constitute the “Trifecta” score. Worrisome is that the majority of the men at risk for poor QOL outcomes are excluded from the Trifecta, leading to inflated “excellent” and misleading results for the “average” men considering RARP treatment. A second problem calls into question the validity of whether men truly equate QOL outcomes truly equal in value to survival. Most importantly, PSA progression free rates decline with time, as noted in Menon et al., BCR-free rates from 95%, 91%, at years 1, 3, to 87%, 81% at 5, 7 years [69]. Hence, Trifecta overstates success at 1-2 years as BCR rates deteriorate with time. 

## 10. Outcomes Self-Assessment

It is imperative for the robotic surgeon to establish a surgical database of preoperative demographics and postoperative outcomes for critical self-evaluation [130]. Self-assessment is a continual iterative process, and as the volume of cases increases, a personal database allows one to quickly measure themselves against published results. Through the process of self-assessment of outcomes, the surgeon can decide if there are specific troublesome technical or clinical issues. There are 2 important self-assessment tools, video recording of cases and rigorous data collection. 

An early and pervasive advantage of RARP, carried over from laparoscopic RP, was the ability to video capture the entire surgery via the 3D camera. Digitally recording each case can be extremely advantageous to not only the novice surgeon but those with experience as well. Reviewing the operation in real-time is particularly useful for difficult cases, complications and PSM. Also, cases with excellent functional outcomes can be reviewed for positive reinforcement of successful techniques, as well as reviewing video-taped footage of one's surgical performance can have a positive impact on improving and evolving surgical technique. 

Video recording had a 2nd more important consequence, the widespread availability of new surgical techniques for incorporation and training across the globe. The visualization of RARP was rapidly disseminated by conferences and scientific meetings and made for quick evaluation by surgeons whether to incorporate changes to their RARP practice or not. For novice surgeons, this was most invaluable.

The robotic interface has also spawned new simulation training methods. Studies have shown that trainees can significantly improve their proficiency on RARP via simulators. New computer-based “virtual” trainers have also been developed and are an attractive training model for the future, if the costs are restrained [131]. 

Data collection of patient demographics and outcomes is also essential for truly understanding the success or failure of robotic surgery. A proposed minimum data collection design is shown in Table 1 [130]. Preoperative data must be stringently collected as most functional outcomes may be dependent on the baseline characteristics of the patient. Preoperative data also gives the surgeon a benchmark, as the ideal goal is to restore all men to preoperative functional status. Postoperative oncologic and functional data, in addition to complication rates, must be meticulously recorded if one is to absolutely critical of their technique and improve surgical performance. If RARP is truly the advance it advocates, then validated questionnaires and analog assessment scales are essential to reliably report true functional results. This is especially difficult in long-term followup of oncologic and quality of life outcomes.

The snag of statistical analyses should be reviewed early on through consultations with an expert statistician. This long-term relationship will make the proposed data collection more effective and efficient. As sexual potency and continence outcomes will not be available until 6–12 months into the surgeon's RARP experience, preconsultation with a statistician regarding data collection methods is truly a strategic and time saving advantage for any program.

## 11. Robotic-Assisted Prostatectomy: Surgical Evolution or New Paradigm

 Has RARP brought incremental changes or quantum leaps? Clearly, RARP has brought new economic costs to the standard radical prostatectomy. However, the long-term cancer and quality of life outcomes are promising. Beyond its general acceptance in many parts of the globe, RARP must always prove that it is a technique that refines radical prostatectomy to belay the additional costs, if it is to become the next evolutionary step.

## 12. Conclusion

 With the introduction of the da Vinci surgical system, we are witnessing a technique shift from open to laparoscopic robotic radical prostatectomy as the procedure of choice at many centers worldwide. While compared to the open approach, early studies indicate that robotic prostatectomy has promising outcomes in short-term oncological control, potency, and continence compared to open radical prostatectomy [22–24]. However, RARP is now beyond its infancy, and the long-term report cards are coming in. Indeed, current results of experienced urologic oncologists with open radical prostatectomy have set high standards in oncologic and functional outcomes. 

 In light of the present day open radical prostatectomy, in order to determine the true place of robotics in the surgical pantheon, validated questionnaires and analog assessment scales are essential to determine true functional results and need to be combined with careful long-term followup of oncologic outcomes. 

 The important paradigm shift introduced by RARP is the *reevaluation of the entire open RP experience *in surgical technique by minimizing blood loss and complications, maximizing cancer free outcomes, and a renewed assault in preserving quality of life outcomes by many novel mechanisms. RARP provides a new technical “canvas” for surgical masters to create upon, and in ten years, has reinvigorated a 100 year old “gold standard” surgery, as Walsh had done 30 years ago.

## Figures and Tables

**Box 1:**
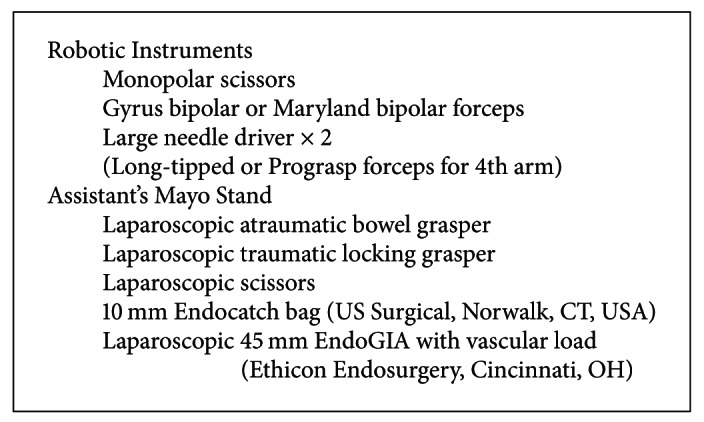
Standard equipment for RLP.

**Box 2:**
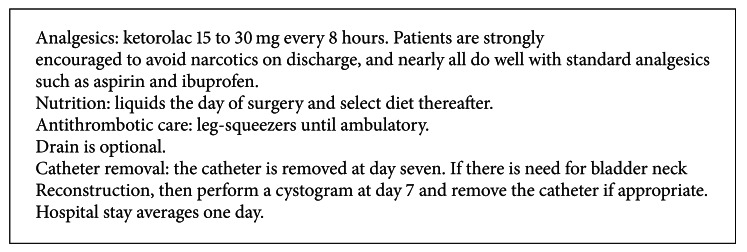
Postoperative care.

**Table 1:** Reduction of major/minor complications through specific interventional techniques, Liss et al. [49].

Complications	Clavien	Old rate	Resolution	New rate	Adjusted *P* value*
Corneal abrasion	1	4/200	Foam-based safety goggles	1/800	0.030
		(2.0%)		(0.1%)	
Fossa strictures	3 a	10/165	Avoidance ≥ 20 F catheters	1/835	0.031
		(6.1%)		(0.1%)	
Bladder neck contractions (BNC)	3 a/3 b	6/592	Addition of “Rocco” Stitch to Van Velthoven single knot anastomosis	1/408	0.052
		(1.0%)		(0.2%)	
Camera site hernias	3 b	40/735	Transverse Incision	1/265	<0.001
		(5.4%)		(0.4%)	
Pulmonary embolus	4 a	5/190	Thigh-high pneumatic compression early and persistent ambulation	0/810	0.863
		(2.6%)		(0%)	

*Adjusted for age, BMI, and surgeon learning curve.

**Table 2:** Univariate and multivariate Cox proportional Hazards regression for long-term risk of BCR-free progression after RARP, Liss et al. [53].

	Univariate				Multivariate*			
Variable		95% CI				95% CI		
	Hazard ratio	Lower	Upper	*P* value	Hazard ratio	Lower	Upper	*P* value
Age	1.01	0.97	1.04	0.663	0.98	0.94	1.01	0.181
PSA	1.09	1.07	1.12	<0.0005	1.07	1.03	1.11	0.0004
Positive margins	4.15	2.64	6.99	<0.0005	1.71	0.98	2.97	0.059
Pathologic Gleason								
≤6	1.00				1.00			
=3 + 4	4.98	2.01	12.35	0.001	3.58	1.40	9.13	0.008
=4 + 3	16.98	6.57	43.83	<0.0005	6.32	2.21	18.04	0.001
≥8	30.10	12.30	73.60	<0.0005	12.24	4.60	32.61	<0.0005
Pathologic stage*								
pT2	1.00				1.00			
pT3A	7.91	4.39	14.23	<0.0005	3.14	1.60	6.14	0.001
pT3B	15.84	8.08	31.06	<0.0005	4.25	2.00	9.03	0.0002

*Excludes 2 patients with pT4.
